# Supplementary material for: Heat-Killed Lactobacilli Preparations Promote Healing in the Experimental Cutaneous Wounds
Source: Cells. 2021 Nov 22;10(11):3264. doi: 10.3390/cells10113264 (PMC8625647; doi:10.3390/cells10113264)
Supplement: Supplementary file 1 [file cells-10-03264-s001.zip › cells-1451440-supplementary.pdf]

Supporting Information

Table S1. The induction of collagen secretion by Hs68 cells with different Lactobacilli.

Species	Strain	mean ± SEM[a]
L.acidophilus	BCRC910774	2.79 ± 0.13
L.acdiophilus	GMNL-631	3.33 ± 0.10
L. helveticus	GMNL-440	5.45 ± 0.04
L. fermentum	BCRC910259	4.28 ± 0.09
L. fermentum	GMNL-297	3.61 ± 0.22
L. fermentum	GMNL-299	3.93 ± 0.03
L. casei	BCRC910585	3.00 ± 0.23
L. paracasei	BCRC910220	4.61 ± 0.15
L. paracasei	BCRC910314	5.32 ± 0.39
L. paracasei	**GMNL-653, BCRC910721**	**5.99 ± 0.02**
L. plantarum	**GMNL-6, BCRC910777**	**9.39 ± 0.27**
L. plantarum	BCRC911066	2.11 ± 0.01
L. pentosus	BCRC910591	3.70 ± 0.10
L. latis	GMNL-480	3.82 ± 0.04

[a] Data were presented as fold change compared with vehicle control.

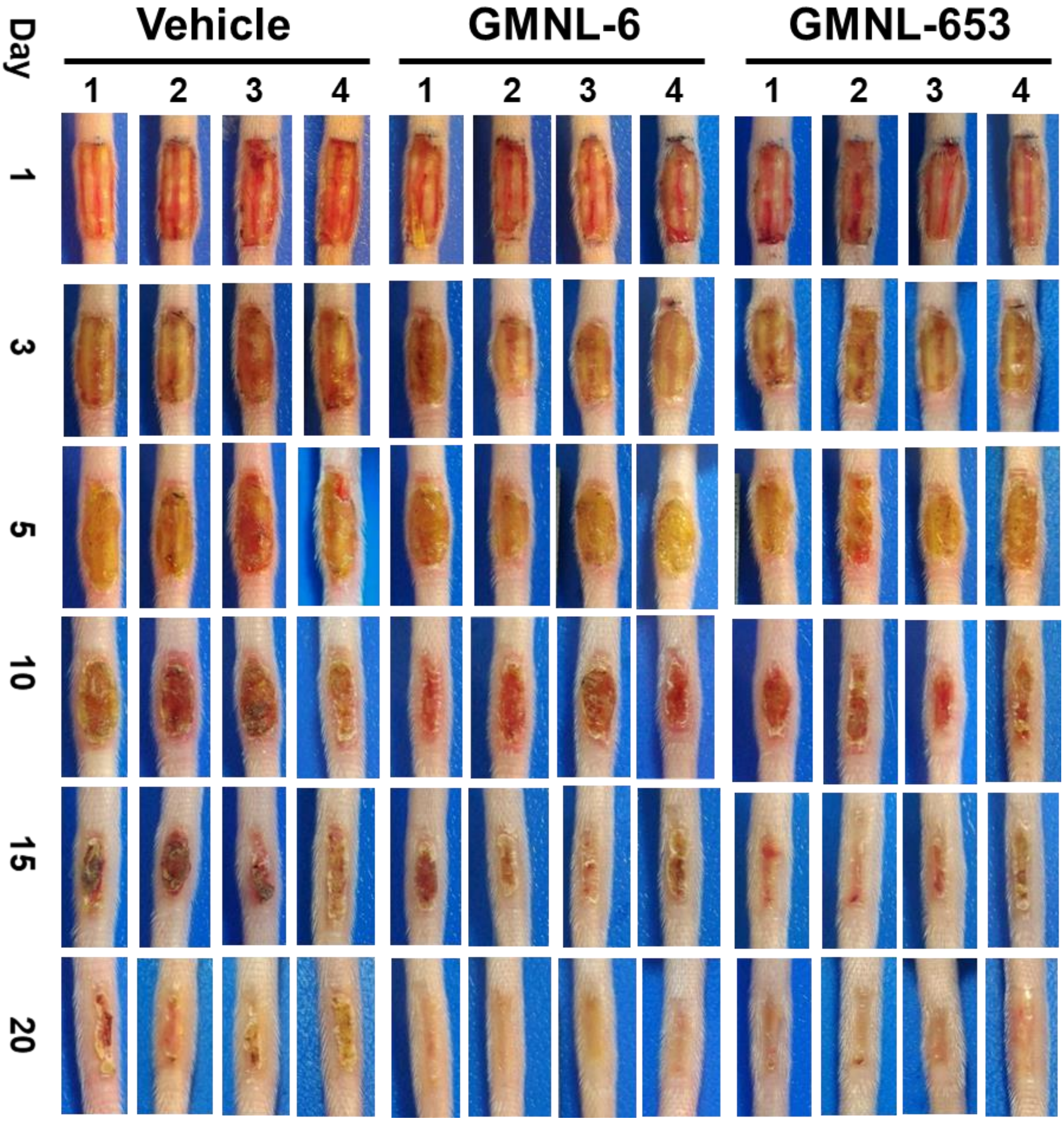

Figure S1. The images of tail wounds from each mouse used for analyzing the beneficial effects of heat-killed GMNL-6- or GMNL-653-containing gels.

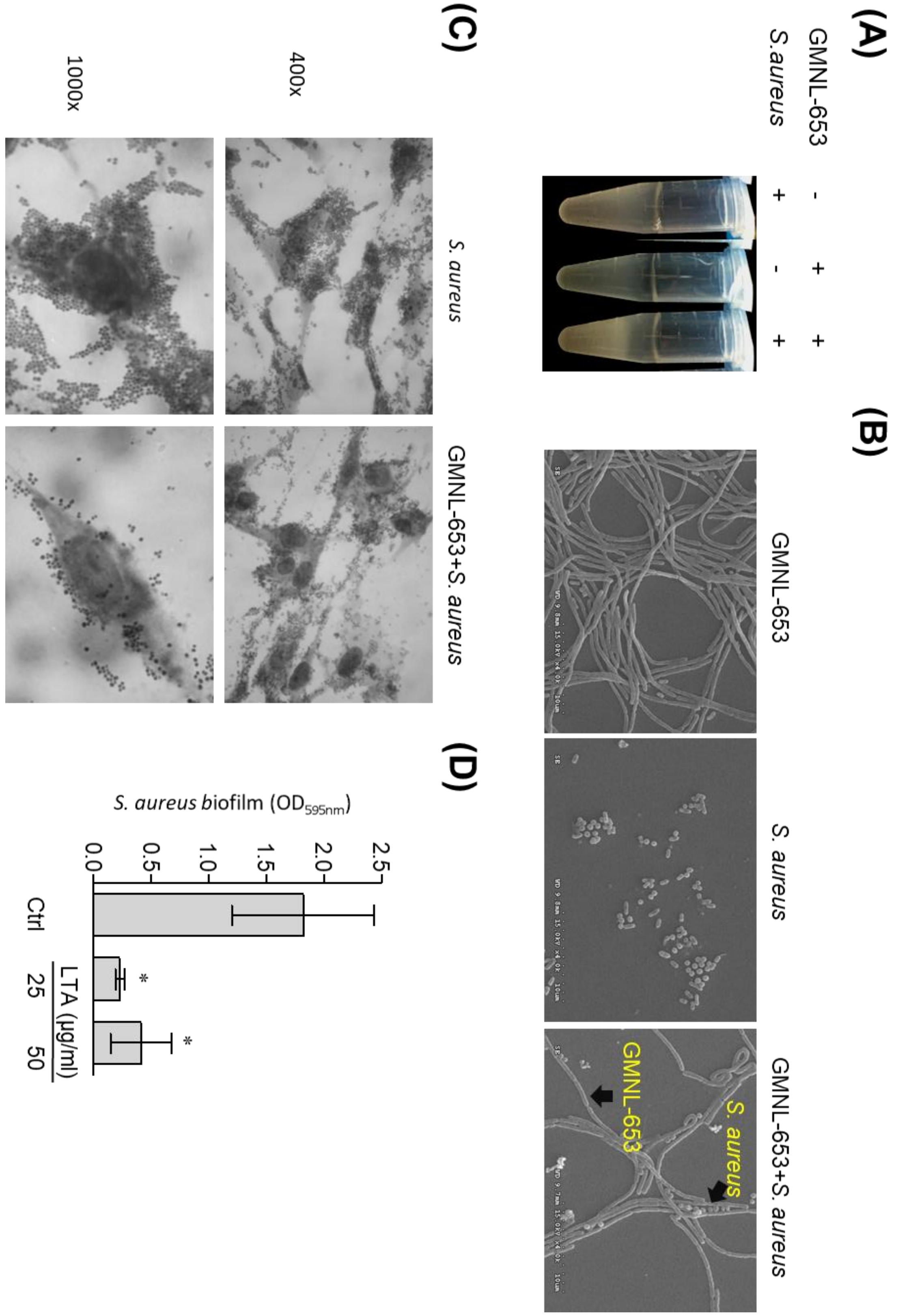

Figure S2. Heat-killed GMNL-653 decreases the adherence of *S. aureus* to Hs68 cells through the induction of aggregation. (A) The tube test of aggregation by directly mixing and observing the formation of precipitates. (B) The SEM data of

aggregates of heat-killed GNML-653 bacterial bodies and live *S. aureus* cells. (C) The live *S. aureus* or the mixture of heat-killed GMNL-653 and live *S. aureus* (1:1) were added into microwells with Hs68 cells for 24 hours followed by observing under microscopy. (D) *S. aureus* were seeded into wells of 96-well-plate with or without the indicated concentration of GMNL-653 derived LTA for 24 hours and the wells were washed with PBS. The biofilm was visualized by staining with 0.1% crystal violet, dissolved with DMSO, and quantified by the absorbance at a wavelength of 590 nm. *, $p < 0.05$.
